# Population Genetics of the Rubber-Producing Russian Dandelion (*Taraxacum kok-saghyz*)

**DOI:** 10.1371/journal.pone.0146417

**Published:** 2016-01-04

**Authors:** Edward V. McAssey, Ethan G. Gudger, Matthew P. Zuellig, John M. Burke

**Affiliations:** 1 University of Georgia, Department of Plant Biology, Miller Plant Sciences Building, Athens, GA 30602, United States of America; 2 University of Georgia, Department of Genetics, Davidson Life Sciences Building, Athens, GA 30602, United States of America; United States Department of Agriculture, UNITED STATES

## Abstract

The Russian dandelion, *Taraxacum kok-saghyz* (TKS), is a perennial species native to Central Asia that produces high quality, natural rubber. Despite its potential to help maintain a stable worldwide rubber supply, little is known about genetic variation in this species. To facilitate future germplasm improvement efforts, we developed simple-sequence repeat (SSR) markers from available expressed-sequence tag (EST) data and used them to investigate patterns of population genetic diversity in this nascent crop species. We identified numerous SSRs (1,510 total) in 1,248 unigenes from a larger set of 6,960 unigenes (derived from 16,441 ESTs) and designed PCR primers targeting 767 of these loci. Screening of a subset of 192 of these primer pairs resulted in the identification of 48 pairs that appeared to produce single-locus polymorphisms. We then used the most reliable 17 of these primer pairs to genotype 176 individuals from 17 natural TKS populations. We observed an average of 4.8 alleles per locus with population-level expected heterozygosities ranging from 0.28 to 0.50. An average pairwise F_ST_ of 0.11 indicated moderate but statistically significant levels of genetic differentiation, though there was no clear geographic patterning to this differentiation. We also tested these 17 primer pairs in the widespread common dandelion, *T*. *officinale*, and a majority successfully produced apparently single-locus amplicons. This result demonstrates the potential utility of these markers for genetic analyses in other species in the genus.

## Introduction

Worldwide natural rubber production is largely reliant on the rubber tree, *Hevea brasiliensis*. Most *H*. *brasiliensis* cultivation occurs in equatorial regions of Southeast Asia, though there is some production in the Amazon basin [1,2]. Unfortunately, this economically important tree species is threatened by South American leaf blight (SALB) [3]. Though progress has been made in understanding the genetic basis of SALB resistance in *H*. *brasiliensis* [4,5], the development of resistant cultivars remains an ongoing challenge [6]. Moreover, there is concern that the responsible pathogen (*Microcyculs ulei*), which is spread by human activities as well as natural spore dispersal [7], will eventually reach Asian rubber tree populations. There is thus great interest in developing alternative sources of natural rubber.

*Taraxacum kok-saghyz* (TKS; Russian dandelion) is a perennial, diploid species that produces large quantities of rubber throughout the plant, especially in its roots. Initial attempts at developing TKS into a viable source of natural rubber were prompted by limited access to rubber plantations during World War II [8]. When the war ended and rubber trees once again became an available source of latex, however, TKS improvement efforts were abandoned. Recent years have seen a renewed interest in the possible development of TKS as a rubber crop, though improvement efforts have been limited in part by a lack of genetic information. While great strides have been made in our understanding of rubber production in TKS and congeners [9–14], we still know very little about the population genetics of this species.

Here, we report the development of simple-sequence repeat (SSR) markers derived from available TKS expressed-sequence tags (ESTs) and their use in population genetic analyses of wild-collected TKS accessions. These EST-SSRs represent the first published nuclear sequence-tagged site (STS) markers for this fascinating species, and their use provides novel insights into the distribution of genetic variation within and among natural populations of TKS.

## Materials and Methods

### EST-SSR discovery and primer design

A total of 16,441 ESTs obtained from the root tissue of young (1 to 3 month) TKS seedlings were downloaded from GenBank (accession numbers DR398435 to DR403165 and GO660574 to GO672283). Sequences were then assembled into contigs using CAP3 [15] with the default settings prior to SSR identification and primer design. The resulting unigene set (i.e., contigs and singletons) was imported into BatchPrimer3 [16] for SSR discovery and primer design. We specifically searched for SSRs using the following repeat criteria: di-nucleotide motif > 5 repeat units; tri-nucleotide motif > 3 repeat units; and quad-, penta- and hexa-nucleotide motifs > 2 repeat units. We next used EST-SCAN [17] and BLAST searches on NCBI to determine whether a given SSR was located in coding sequence (CDS) or an untranslated region (UTR). PCR primer design was performed using the default settings. A subset of 192 primer pairs was ordered for testing (Integrated DNA Technologies, Coralville, IA, USA). Notably, a 19 bp extension (corresponding to the M13 forward primer sequence) was added to the 5’ end of every forward primer to facilitate the use of a three-primer protocol for SSR amplification and fluorescent labeling [18,19], as described below.

### DNA extraction and quantification

Seeds derived from 17 wild-collected TKS populations in Kazakhstan were obtained from the USDA-ARS Western Regional Plant Introduction Station. Seeds from each population were placed on moist filter paper and stored in darkness at 4C for 48 hours before being allowed to germinate at room temperature. Following germination, seedlings were transplanted into pots in the University of Georgia greenhouses (Athens, GA, USA). Once established, 600 mg of fresh tissue was collected from each individual for DNA extraction using the Qiagen (Valencia, CA, USA) DNeasy Plant Mini Kit (176 individuals total from 17 populations; mean = 10.4 individuals per population, range = 7–16). Following extraction, DNA was quantified using a NanoDrop (Thermo Fisher Scientific, San Diego, CA, USA) and diluted to 2.5 ng/μl in preparation for genotyping. We also collected tissue and extracted DNA (as above) from four individuals of *T*. *officinale* collected from Athens, GA to facilitate an investigation of the transferability of our markers to other species within the genus.

### EST-SSR genotyping

All genotyping was done using a modification of the three-primer PCR protocol described by Schuelke [18], as adapted by Wills et al. [19]. We pre-screened the full set of 192 primer pairs described above on a test panel of eight TKS individuals, all from different populations, plus four *T*. *officinale* individuals. Reactions were performed in a total volume of 15 μL containing 2.5 ng of template DNA, 30 mM Tricine pH 8.4-KOH, 50 mM KCl, 2 mM MgCl_2_, 125 μM of each dNTP, 0.1 μM reverse primer, 0.02 μM forward primer, 0.1 μM fluorescently labeled M13 primer (either FAM, NED or HEX), and 1 unit of *Taq* polymerase. The PCR conditions were as follows: 3 min at 95C; 10 cycles of 30 s at 94C, 30 s at 65C and 45 s at 72C, annealing temperature decreasing to 55C by 1C per cycle, followed by 30 cycles of 30 s at 94C, 30 s at 55C, 45 s at 72C, followed by 20 min at 72C. PCR products were visualized on 1% agarose gels to identify primers that successfully produced amplicons. If the above cycling conditions were unsuccessful at producing a single-banded amplicon in TKS, the annealing temperature was increased or decreased, as appropriate, to achieve more or less stringent reaction conditions. For primer pairs that produced a single-banded amplicon, the reactions were diluted 1:15 and visualized on an ABI 3730xl (Applied Biosystems, Foster City, CA, USA) with GGF 500 ROX size standard (Georgia Genomics Facility, Athens, GA, USA), which includes standards ranging in size from 88 to 435 bp. Data files were imported into GeneMarker (SoftGenetics, State College, PA, USA; version 1.85) for genotype calling. The trace files were manually inspected to ensure accurate genotyping and 48 primer pairs that produced readily-interpretable, single-locus polymorphisms in the aforementioned test panel of eight TKS individuals were then used to genotype the full panel of TKS individuals following the same general methods. As more individuals were genotyped, we reduced our final dataset from the original 48 primer pairs to a subset of 17 loci that consistently provided easily interpretable, single-locus polymorphisms. The remaining 31 loci were excluded due to missing data or more complex (i.e., multi-locus) banding patterns that emerged when the larger set of samples was considered.

### Population genetic analyses

The multi-locus genotype data were analyzed using GenAlEx (version 6.501) [20]. For each population, we calculated the observed heterozygosity, Nei’s unbiased expected heterozygosity [21], and Wright’s fixation index [22]. Additionally, F_ST_ and G_ST_ were calculated for each locus. An analysis of molecular variation (AMOVA) was also performed in GenAlEx using 999 permutations to determine the amount of genetic variation that is significantly attributable to populations. We performed a principal coordinates analysis (PCoA) by using a standardized genetic distance matrix as calculated in GenAlEx. A Mantel test was performed to test the correspondence between a pairwise geographic distance matrix (and its log transformation) and a pairwise Nei’s genetic distance matrix among all 17 populations.

## Results and Discussion

### Identification and characterization of EST-SSRs

Assembly of the 16,441 TKS ESTs resulted in a unigene set of 6,960 contigs and singletons. Of these, a total of 1,248 (17.9%) were found to contain at least one SSR, on par with the values from other plant species [23]. Of the 1,248 SSR-containing contigs and singletons, 154 contained 2 SSRs, 40 contained 3 SSRs, 8 contained 4 SSRs, and 1 contained 5 SSRs, for a total of 1,510 SSRs. As has been observed in other species [24–29], we found that the majority of EST-SSRs (55.0%) were tri-nucleotide repeats, likely due to the fact that such repeats will not disrupt the reading frame when length variants occur in coding regions [23] (Table 1; S1 Fig).

**Table 1:** SSR abundance and length by repeat size. Relevant summary statistics are presented for each size class of repeat motif.

	Di-	Tri-	Quad-	Penta-	Hexa-
Number of SSRs	213	830	237	70	160
Mean number of repeats per SSR	9.69	4.73	3.21	3.19	3.35
Range of repeat number per SSR^1^	6–30	4–14	3–9	3–5	3–7

^1^ The lower limit of repeat number for each size class was dictated by the parameters used during SSR identification. For Di-, Tri-, Quad-, Penta- and Hexa- the lower limit for repeat detection was 6, 4, 3, 3 and 3, respectively.

**Fig 1:**
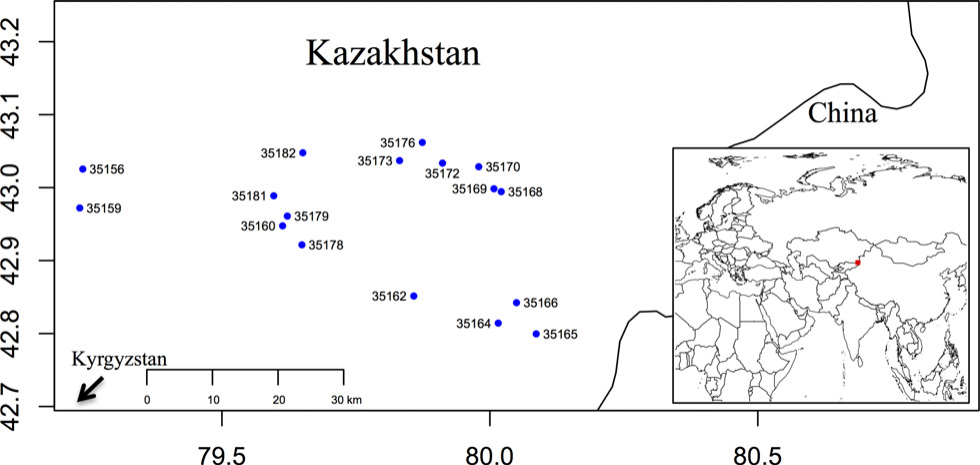
Map indicating the locations of the 17 sampled Russian dandelion populations. Blue dots represent the locations of the populations. The x- and y-axes represent longitude and latitude, respectively. The main and inset maps were made using the R libraries ‘maps’ and ‘maptools’, respectively [30].

To investigate the utility of these EST-SSRs for population genetic analyses, we tested a subset of 192 pairs on a panel of one Russian dandelion individual derived from each of eight wild populations in Kazakhstan. These 192 primer pairs targeted 70 di-nucleotide repeats, 119 tri-nucleotide repeats, and 3 quad-nucleotide repeats; all of these primer pairs targeted EST-SSRs with at least 5 repeat units in the source EST. Of these tested primer pairs, 48 initially produced single-locus, polymorphic amplicons from a majority of test samples. However, as mentioned above, a subset of 17 primer pairs were found to be the most reliable and were used across the full collection of individuals. Ultimately, we genotyped 176 TKS individuals from 17 natural populations (average = 9.9 individuals genotyped per population per locus after accounting for missing data) in southeast Kazakhstan near the border with western China (Fig 1). These 17 loci were used in our population genetic analyses (details below), and their primers were also tested on 4 individuals from *T*. *officinale* to assess cross-taxon transferability. Twelve of these seventeen primer pairs successfully produced single-banded amplicons in *T*. *officinale* (Table 2), demonstrating the potential utility of these markers for analyses in other species within the genus.

**Table 2:** Results of cross-amplification of 17 newly developed SSRs in *T*. *officinale*.

Locus	Amplified	Single banded
TKS_0003	+	+
TKS_0025	+	+
TKS_0085	+	-
TKS_0091	+	+
TKS_0097	+	-
TKS_0105	+	+
TKS_0107	+	+
TKS_0110	+	+
TKS_0111	+	+
TKS_0112	+	+
TKS_0113	+	-
TKS_0114	+	+
TKS_0123	+	-
TKS_0134	+	-
TKS_0138	+	+
TKS_0149	+	+
TKS_0177	+	+

### Population genetic diversity

The mean number of alleles per locus was 4.8 ± 0.6 (mean ± SE; range 2–11) across the full TKS dataset (i.e., all 176 individuals from 17 populations; Table 3). Levels of allelic variability showed some general trends with respect to the location of SSRs within ESTs. Notably, SSRs in untranslated regions had an average of 5.3 ± 1.2 alleles while those in coding sequences had an average of 4.5 ± 0.5 alleles, though this difference was not statistically significant (*P* > 0.05). Similar to our results, Gupta et al. [24] found that SSRs located in UTRs tended to be more polymorphic than those found in coding sequence (CDS) in bread wheat, perhaps due to more stringent selection against length variants in coding regions. In contrast, results from *Helianthus* suggest that SSRs found in CDS vs. UTRs exhibit similar levels of polymorphism [31].

**Table 3:** Summary statistics for EST-SSR variation in natural Russian dandelion populations. CDS = Coding sequence, 5’ UTR = 5’ untranslated region, 3’ UTR = 3’ untranslated region, N = number of individuals successfully genotyped out of 176, N_A_ = total number of alleles.

TKS ID	Location	N	N_A_	SSR Motif	Allele size range	F_ST_	G_ST_	F_IS_
TKS_0003	5' UTR	169	5	AG	189–203	0.12*	0.06*	0.20
TKS_0025	5' UTR	166	2	TCA	281–284	0.08	0.02	-0.01
TKS_0085	CDS	163	8	ATC	150–174	0.11*	0.03*	0.48
TKS_0091	3' UTR	151	4	TA	168–183	0.11*	0.05*	-0.09
TKS_0097	3' UTR	175	4	AT	159–165	0.08	0.02	0.13
TKS_0105	3' UTR	173	3	AGA	173–179	0.14*	0.09*	0.04
TKS_0107	CDS	163	6	GCT	247–252	0.19*	0.11*	0.62
TKS_0110	CDS	174	5	GGA	168–180	0.13*	0.08*	0.03
TKS_0111	CDS	170	3	TCG	178–184	0.12*	0.08*	-0.18
TKS_0112	3' UTR	170	11	ATC	141–164	0.12*	0.07*	0.02
TKS_0113	CDS	168	5	AAC	141–153	0.14*	0.10*	0.01
TKS_0114	CDS	169	3	GAT	165–171	0.12*	0.06*	0.05
TKS_0123	CDS	173	5	TGA	154–169	0.12*	0.07*	0.19
TKS_0134	3' UTR	163	8	TCT	172–203	0.10*	0.05*	-0.11
TKS_0138	CDS	174	2	TCC	163–166	0.11*	0.06*	-0.11
TKS_0149	CDS	174	4	GCC	176–185	0.10*	0.06*	-0.19
TKS_0177	CDS	161	4	GAA	192–204	0.06	0.01	-0.12
Average		168	4.8			0.11	0.06	0.06

* *P* **≤** 0.05.

Averaging across the 17 loci, the observed heterozygosity levels within populations ranged from 0.28 to 0.47 (0.37 ± 0.01), whereas unbiased expected heterozygosity levels ranged from 0.28 to 0.50 (0.43 ± 0.01; Table 4). After correcting for multiple tests, only three population/locus combinations were significantly different from Hardy-Weinberg expectations (Bonferroni-adjusted *P* < 0.05). Furthermore, we found only one population-level F_IS_ value that was significantly different from zero (all others *P* > 0.05). This particular population (35169) had a significantly positive F value overall, though it had negative F_IS_ values (higher observed heterozygosity relative to expected heterozygosity) for six of the sixteen loci with F_IS_ values (one locus was monomorphic in this population). Two loci in population 35169 had exceptionally elevated F_IS_ estimates compared to the average across loci, which may indicate the presence of population specific null alleles, or may be the result of selection. As a whole, these results suggest that there are little or no systemic deviations from Hardy-Weinberg equilibrium within the other 16 populations, though it should be noted that average sampling per population was somewhat limited. It is thus possible that deeper sampling would reveal significant deviations that previously escaped detection. Within populations, the mean percent polymorphic loci was 94.5% ± 1.6% and contained one outlier value of 70.6%.

**Table 4:** Summary of population genetic diversity statistics. Values averaged across 17 loci for each of the 17 populations. N = number of individuals genotyped, H_o_ = observed heterozygosity, UH_e_ = unbiased expected heterozygosity, F = Wright’s fixation index.

Population		N	H_o_	uH_e_	F
35156	Mean	8.9	0.40	0.49	0.13
	SE	0.4	0.04	0.04	0.08
35159	Mean	7.1	0.47	0.50	-0.01
	SE	0.3	0.06	0.04	0.11
35160	Mean	15.4	0.34	0.41	0.09
	SE	0.2	0.06	0.06	0.08
35162	Mean	8.6	0.29	0.28	-0.07
	SE	0.2	0.07	0.05	0.10
35164	Mean	8.7	0.47	0.48	-0.08
	SE	0.1	0.06	0.05	0.10
35165	Mean	12.5	0.38	0.41	0.01
	SE	0.2	0.06	0.04	0.09
35166	Mean	9.7	0.35	0.39	0.01
	SE	0.1	0.05	0.04	0.08
35168	Mean	10.8	0.38	0.45	0.09
	SE	0.1	0.05	0.04	0.09
35169	Mean	11.5	0.34	0.44	0.15*
	SE	0.2	0.05	0.05	0.07
35170	Mean	11.4	0.32	0.39	0.17
	SE	0.2	0.05	0.04	0.09
35172	Mean	6.5	0.39	0.47	0.09
	SE	0.3	0.06	0.04	0.11
35173	Mean	9.8	0.41	0.46	0.05
	SE	0.1	0.06	0.05	0.09
35176	Mean	10.5	0.36	0.45	0.13
	SE	0.2	0.04	0.04	0.07
35178	Mean	6.7	0.38	0.45	0.04
	SE	0.2	0.05	0.04	0.09
35179	Mean	10.8	0.28	0.34	0.07
	SE	0.2	0.05	0.05	0.07
35181	Mean	9.8	0.42	0.46	0.02
	SE	0.1	0.05	0.04	0.07
35182	Mean	9.5	0.36	0.40	0.04
	SE	0.2	0.05	0.05	0.06

*P **≤** 0.05.

A number of previous studies have used molecular markers (including SSRs) to investigate genetic diversity in wild *Taraxacum* populations. For example, various marker types (including SSRs) have been used to assess levels of diversity in asexual and sexual dandelion populations in central Europe [32]. Additionally, population genetic markers have been used to describe the relative contributions of mutation and recombination in generating genotypic diversity in *T*. *officinale* [33]. In TKS, amplified fragment length polymorphisms (ALFPs) have been used differentiate TKS individuals from another species of dandelion, *T*. *brevicorniculatum* [34]. Specifically, the researchers show that within species genetic variation is much higher in TKS compared to the apomitic *T*. *brevicorniculatum* [34]. While their within population sample size precluded a direct calculation of population structure for TKS, a principal coordinates analysis coupled with a dendrogram both suggested relatively low levels of population structure in accordance with our results [34].

### Population genetic structure

In terms of population structure, the average F_ST_ across all 17 loci, and considering all 17 populations, was 0.11 ± 0.007 (mean G_ST_ was 0.06 ± 0.007). Levels of pairwise F_ST_ in all population comparisons were also relatively low (S2 Table). In addition to the low average level of differentiation amongst these populations, a Mantel test revealed no signature of isolation-by-distance in our dataset (*P* > 0.05). These results are generally consistent with the expectation for plant species with wind-based seed dispersal. Indeed, Nybom [35] found an average F_ST_ level of 0.13 for such species in a meta-analysis of SSR-based studies compared to species with ingested seeds (F_ST_ = 0.21), attached seeds (F_ST_ = 0.33), and gravity-dispersed seeds (F_ST_ = 0.34) (all F_ST_ estimates from [35]). Additionally, there exists a trend toward long-lived perennials (F_ST_ = 0.19) having less genetic structure relative to both short-lived perennials (F_ST_ = 0.31) and annuals (F_ST_ = 0.40) [35]. However, genetic structuring need not be solely attributed to seed dispersal and life history. Indeed, when comparing average F_ST_ of species with different breeding systems, it has been shown that both selfing species (F_ST_ = 0.42) and those with mixed mating systems (F_ST_ = 0.26) exhibit higher levels of differentiation compared to outcrossing species (F_ST_ = 0.22) [35]. An earlier meta-analysis of population genetic studies using allozymes also revealed that, for outcrossing species, genetic structure was lower for wind-dispersed species (G_ST_ = 0.101) when compared to species with seeds dispersed via animal ingestion (G_ST_ = 0.223) or gravity (G_ST_ = 0.189), but was not statistically different from species with animal-attached seeds (G_ST_ = 0.114) [36].

**Fig 2:**
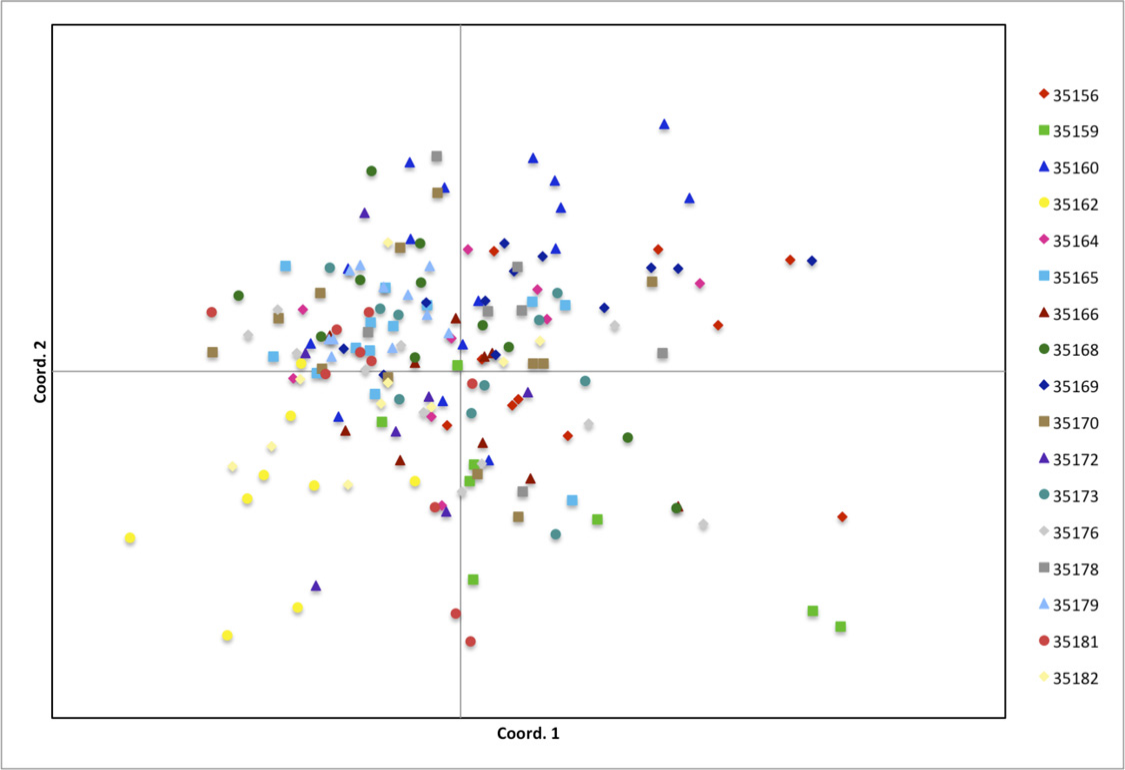
Principal coordinates analysis (PCoA) based on a genetic distance matrix of individuals within the 17 sampled populations. The first two coordinates explain 9.27% and 7.90% of the observed variation, respectively.

Our PCoA further indicated a general lack of structuring with the first axis explaining 9.3% of the variation and the second axis explaining an additional 7.9% (Fig 2). Moreover, the PCoA did not reveal any population that was particularly well-differentiated from the rest; rather, our results suggest a history of substantial gene flow amongst populations. In general agreement with our F_ST_ and PCoA analyses, an analysis of molecular variance (AMOVA) attributed only 7% (*P* = 0.001) of the genetic variance to population-level differentiation. This relatively low level of genetic structure could be a byproduct of relatively high levels of seed and/or pollen dispersal. Further insight awaits direct investigation of pollen vs. seed-mediated rates of gene flow (e.g., [37]). Relatively low values of genetic structure suggest that most of the genetic variation in TKS can be found within populations. Given these results, deep sampling of relatively few populations may be sufficient to capture the majority of the genetic diversity present within this species.

## Conclusions

This study has resulted in the development of a set of well-characterized, polymorphic genetic markers for use in TKS, along with additional, untested PCR primer pairs for the potential development of additional markers. The majority of these markers were found to produce apparently single-locus amplicons in a related species, indicating their potential utility for investigating patterns of genetic variation in other species within the genus. Moreover, our work has provided valuable insight into patterns of genetic diversity in wild populations sampled from throughout the native range of this prospective industrial crop species. The apparently low level of population genetic structure, which may be attributable to potentially high levels of seed and/or pollen dispersal, suggest that much of the neutral genetic diversity within Russian dandelion resides within populations, and can thus be captured by targeted sampling from a subset of the species range. The extent to which these populations vary in terms of adaptively important genetic variation remains to be seen.

## Supporting Information

S1 Fig Distribution of repeat number for each repeat size class. The x-axis indicates the number of repeat units.
(TIFF)

S1 Table Primer sequences for the 17 SSRs used for population genetic analyses (first tab), the non-selected set of 175 SSRs (second tab), and a set of 95 untested SSRs (third tab). (XLSX)

S2 Table Pairwise F_ST_ estimates among 17 natural populations of Russian dandelion. Below diagonal values are pairwise F_ST_ and above diagonal values represent the *P* value for the corresponding F_ST_.
(XLSX)

S3 Table SSR genotypes for 176 Russian dandelion individuals. Numbers represent allele sizes in base pairs. Missing data is represented by ‘0’.
(XLSX)
