# Supplementary figures and images for: Population Genetics of the Rubber-Producing Russian Dandelion (Taraxacum kok-saghyz)
Source: PLoS One. 2016 Jan 4;11(1):e0146417. doi: 10.1371/journal.pone.0146417 (PMC4703197; doi:10.1371/journal.pone.0146417)

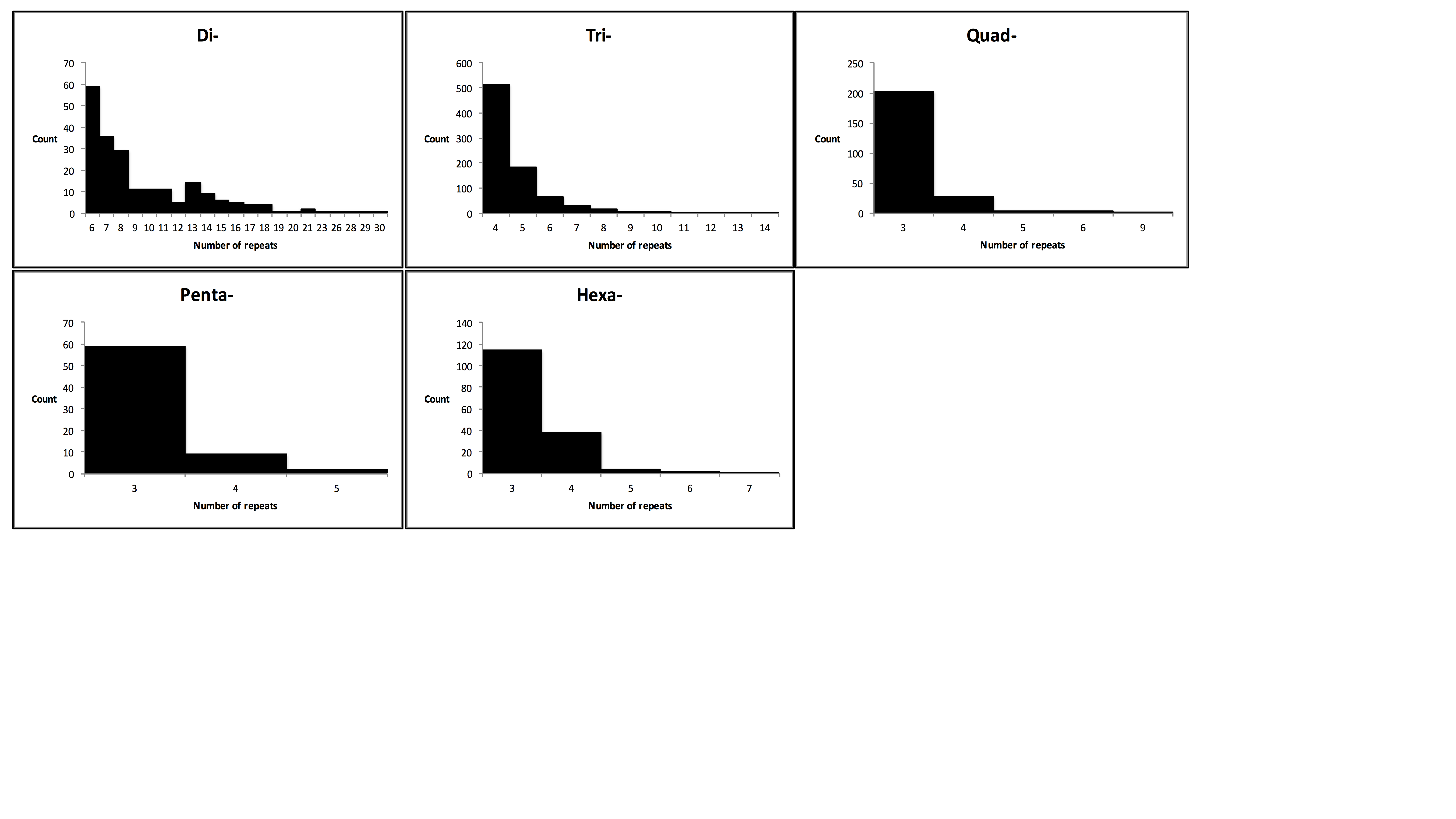

Supplement: S1 Fig — The x-axis indicates the number of repeat units. (TIFF) [file pone.0146417.s001.tiff]
